# *Leishmania infantum* Induces Mild Unfolded Protein Response in Infected Macrophages

**DOI:** 10.1371/journal.pone.0168339

**Published:** 2016-12-15

**Authors:** Luca Galluzzi, Aurora Diotallevi, Mauro De Santi, Marcello Ceccarelli, Fabrizio Vitale, Giorgio Brandi, Mauro Magnani

**Affiliations:** 1 Department of Biomolecular Sciences, University of Urbino Carlo Bo, Urbino, Italy; 2 Istituto Zooprofilattico Sperimentale of Sicily A Mirri, Palermo, Italy; Instituto Oswaldo Cruz, BRAZIL

## Abstract

The Leishmaniases are a group of parasitic diseases caused by protozoa of the *Leishmania* genus affecting both humans and other vertebrates. *Leishmania* is an intracellular pathogen able to confer resistance to apoptosis in the early phase of macrophages infection by activation of host PI3K/Akt pathway and inhibition of caspase-3 activation. Intracellular pathogens hijack organelles such as ER to facilitate survival and replication, thus eliciting ER stress and activating/modulating the unfolded protein response (UPR) in the host cell. The UPR is aimed to mitigate ER stress, thereby promoting cell survival. However, prolonged ER stress will activate the apoptotic pathway. The aim of this study was to investigate the ER stress response in *Leishmania*-infected macrophages to gain insights about the mechanisms underlying the apoptosis resistance in parasitized cells. Macrophages differentiated from human monocytic cell lines (U937 and THP-1) and murine primary macrophages were infected with *Leishmania infantum* MHOM/TN/80/IPT1 (WHO international reference strain). Several ER stress/autophagy expression markers, as well as cell survival/apoptosis markers (phospho-Akt and cleaved caspase-3) were evaluated by qPCR and/or by western blotting. As ER stress positive control, cells were treated with tunicamycin or dithiothreitol (DTT). The gene expression analyses showed a mild but significant induction of the ER stress/autophagy markers. The western blot analyses revealed that the *Leishmania* infection induced Akt phosphorylation and significantly inhibited the induction of caspase-3 cleavage, eIF2α phosphorylation and *DDIT3/CHOP* expression in tunicamycin and DTT treated cells. The mild but significant increase in ER stress expression markers and the delay/attenuation of the effects of ER stress inducers in infected cells support the hypothesis that *L*. *infantum* could promote survival of host cells by inducing a mild ER stress response. The host ER stress response could be not only a common pathogenic mechanism among *Leishmania* species but also a target for development of new drugs.

## Introduction

Leishmaniasis is a complex disease caused by numerous species of the protozoan parasite *Leishmania*. Depending on the *Leishmania* species and the characteristics of the host, the infection results in lesions at cutaneous sites or in visceral organs. *Leishmania* promastigotes preferentially infect macrophages in their mammalian hosts. The infection leads to subversion/modulation of the host’s innate immune response and cellular metabolic pathways, thereby allowing parasite survival and replication [1,2]. Notably, parasitized cells become resistant to apoptosis [3–5], therefore the death of infected macrophages is delayed. In particular, Ruhland et al [6] identified the activation of PI3K/Akt pathway as an important pathway engaged by *Leishmania major* and *Leishmania mexicana* complex (*L*. *amazonensis* and *L*. *pifanoi*) to confer host cell resistance to apoptosis.

The endoplasmic reticulum (ER) stress is caused by perturbation of ER functions (*i*.*e*. protein synthesis/folding/post-translational modifications, biosynthesis of lipids and sterols, Ca^2+^ storage). The ER stress response, called the Unfolded Protein Response (UPR), is an evolutionary conserved mechanism aimed to restore ER homeostasis and ensure cell survival. In mammalian cells, the UPR is driven primarily by three ER-transmembrane proteins: inositol-requiring enzyme 1 (IRE1), PKR-like endoplasmic reticulum kinase (PERK) and activating transcription factor 6 (ATF6) [7]. The activation of IRE1 and PERK is mediated by phosphorylation, while activation of ATF6 occurs via proteolytic cleavage after translocation to the Golgi apparatus. The activated IRE1 removes a 26 bp intron from cytoplasmic unspliced X-box binding protein 1 (uXBP1) mRNA [8]. This splicing leads to a frame shift that extends the open reading frame and gives rise to the spliced XBP1 (sXBP1) transcription factor, which induces the expression of several chaperones and proteins involved in ER-associated degradation (ERAD) and/or autophagic response [9]. The uXBP1 acts as a negative regulator of sXBP1, promoting its degradation by proteasome [10]. Further investigations on the role of uXBP1 demonstrated its synergistic action with histone deacetylase 3 (HDAC3) in Akt phosphorylation and NRF2-mediated heme oxygenase 1 (HO-1) expression, to protect endothelial cells from oxidative stress [11]. The activated PERK phosphorylates the alpha subunit of eukaryotic initiation factor 2 (eIF2α), leading to global translation attenuation. At the same time, preferential translation of activating transcription factor 4 (ATF4) is induced. ATF4, in turn, induces the expression of C/EBP-homologous protein (DDIT3/CHOP) and genes involved in antioxidant stress responses [7]. ATF6 contributes to optimization of the UPR by controlling a number of proteins, some of which are regulated also by XBP1 [12]. Together, these three branches contribute to mitigation of ER stress, thereby promoting cell survival. However, if ER stress is prolonged and cannot be reversed, the cell death occurs, mainly by apoptosis [7].

It is known that viruses and other intracellular pathogens hijack the ER in order to facilitate their replication, thus activating the UPR [12]. UPR and innate immune pathways are significantly interconnected and can regulate each other [13]. Therefore, the microorganism-induced alterations of the UPR can influence the immune response. For example, the activation of UPR mediated by viruses can suppress innate antiviral immunity [12]. Moreover, *Plasmodium* infection of hepatocytes is associated with ER stress and UPR [14]; *Mycobacterium tubercolosis* infection leads to ER stress-induced apoptosis [15]; the intracellular parasite *Toxoplasma gondii* promotes the proteasome-mediated degradation of ATF6β in infected cells through the virulence factor ROP18 [16]; *Pseudomonas aeruginosa* induces UPR to facilitate survival of infected host cell [17].

Despite these evidences, little is known about induction and/or modulation of UPR in macrophages infected by the protozoan parasite *Leishmania* and its role in the pathogenesis. In this study, we evaluated the UPR response in human and murine macrophages infected with *L*. *infantum*. The infection elicited a mild ER stress response that delays/attenuates the effects of the ER stressors tunicamycin and DTT, indicating the possible exploitation of UPR to improve survival of infected cells.

## Materials and Methods

### Parasite cultures

The reference strain *Leishmania infantum* MHOM/TN/80/IPT1 (WHO international reference strain) was obtained from the OIE Reference Laboratory National Reference Centre for Leishmaniasis (C.Re.Na.L.) (Palermo, Italy). *L*. *infantum* amastigotes were also isolated from lymph node aspirates of two infected symptomatic dogs, obtained from the veterinary clinic Santa Teresa (Fano, Italy). The dogs were diagnosed using an indirect fluorescent antibody test (IFAT), prepared using whole fixed *L*. *infantum* MHOM/TN/80/IPT1 promastigotes as antigen [18]. Moreover, the diagnosis of *L*. *infantum* infection was confirmed by a real-time PCR assay from conjunctival swabs and lymph nodes [19].

*L*. *infantum* MHOM/TN/80/IPT1 and isolated strains were cultivated at 26–28°C in Evans’ Modified Tobie’s Medium (EMTM), prepared as described previously [20]. Stationary promastigote were transferred to fresh medium (ratio 1:5) every 3 days.

### Cell culture, treatment and infection

The human monocytic cell lines U937 (ATCC CRL-1593.2) and THP-1 (ATCC TIB-202) were cultured in a humidified incubator at 37°C and 5% CO_2_ in RPMI-1640 medium supplemented with 10% heat-inactivated Fetal Bovine Serum (FBS), 2 mM L-glutamine, 10 g/l Non-Essential Amino Acid, 100 μg/ml streptomycin, 100 U/l penicillin (complete medium). To induce differentiation into macrophages-like cells, 6 x 10^5^ cells were seeded in 35 mm dishes and treated with 10 ng/ml phorbol myristic acid (PMA) for 24 h. Then, the medium was replaced with regular complete medium and cells were incubated for a further 48 h.

Murine primary macrophages were removed from seven ICR/CD-1 mice (Harlan Nossan, Milan, Italy) by peritoneal washing and cultured in RPMI-1640 complete medium overnight at 37°C in a humidified 5% CO_2_ atmosphere. Non-adherent cells were removed by gentle washing with warm PBS. The adherent cells were incubated for a further 24 h in RPMI-1640 complete medium before *L*. *infantum* infection. All cell culture reagents were purchased from Sigma-Aldrich (St. Louis, MO).

Differentiated macrophages from U937 and THP-1 cells were infected with stationary promastigotes resuspended in RPMI-1640, at a parasite-to-cell ratio of 10:1. The dishes were centrifuged at 450 x g for 3 min to synchronize the infection. The murine peritoneal macrophages were infected with 5x10^6^ stationary promastigotes per dish. After 6 h, 18 h and/or 24 h cells were washed to remove free parasites and directly lysed for downstream analyses, while one dish was stained with Hoechst dye to monitor infection with a fluorescence microscope. The infection index was calculated by multiplying the percentage of infected macrophages by the average number of parasites per macrophage. At least 300 total macrophages were counted for each time of infection. *Leishmania* parasites infecting macrophages were alternatively quantified using a previously developed qPCR assay with a standard curve established with serial dilutions of *L*. *infantum* MHOM/TN/80/IPT1 DNA [21]. To evaluate the modulation of ER stress response during infection, we treated the cells with vehicle (DMSO) or 0.5 μg/ml tunicamycin for 2–6 h. As alternative ER stress inducer, 1 mM dithiothreitol (DTT) was used.

### Quantitative real-time PCR (qPCR)

Both infected and uninfected cells were direct lysed at different time points with 300 μl buffer RLT (Qiagen, Hilden, Germany). Total RNA was extracted from lysed cells using the RNeasy plus kit (Qiagen, Hilden, Germany), quantified using a NanoVue Plus^™^ spectrophotometer (GE Healthcare Life Sciences, Piscataway, NJ, USA), and reverse transcribed using PrimeScript^™^ RT Master Mix (Perfect Real Time) (Takara Bio Inc., Otsu, Shiga, Japan) according to the manufacturer’s instructions. The integrity of RNA was assessed by 1% agarose gel stained with GelRed (Biotium, Hayward, CA). The qPCR was performed using the primer pairs listed in Tables 1 and 2, for human and murine targets, respectively. All reactions were performed in duplicate in a final volume of 20 μl, using SYBR Green PCR master mix (Diatheva, Italy), 200 nM primers, in a RotorGene 6000 instrument (Corbett life science, Sydney, Australia). The amount corresponding to 50 ng of total RNA used for cDNA synthesis was loaded per each PCR tube. The amplification conditions for all targets, with the exception of human gene *ATF3*, were as follows: 95°C for 10 min, 40 cycles at 95°C for 15 s and 60°C for 50 s. With regard to the human gene *ATF3*, the annealing/extension step was reduced to 40 s and a further step at 84°C for 10 s was added. A duplicate non-template control was included for each primer pair reaction. At the end of each run, a melting curve analysis from 60°C to 95°C was performed to ensure the absence of primer dimers or non-specific products. For human genes, *B2M* (beta-2-microglobulin) and/or *GUSB* (Beta-D-Glucuronidase) were selected as reference genes among four candidates (*B2M*, *GAPDH*, *HPRT1*, *GUSB*). For mouse genes, *B2m* was selected as reference gene between two candidates (*B2m* and *Gapdh*). Relative mRNA expression was calculated using the comparative quantification application of the RotorGene 6000 software.

**Table 1:** Human qPCR primers.

Target mRNA	Accession number	Forward primer (5’-3’)	Reverse primer (5’-3’)	Ref
DDIT3 (CHOP)	NM_001195053	GGAGCATCAGTCCCCCACTT	TGTGGGATTGAGGGTCACATC	[22]
HSPA5	NM_005347	CCCCGAGAACACGGTCTTT	CAACCACCTTGAACGGCAA	[23]
ATF3	NM_001674	GCCATCCAGAACAAGCACCT	GGCTACCTCGGCTTTTGTGAT	[23]
ATF4	NM_001675	CCCTTCACCTTCTTACAACCTC	TGAAGGAGATAGGAAGCCAGAC	
MAP1LC3B	NM_022818	AAACGGGCTGTGTGAGAAAAC	TGAGGACTTTGGGTGTGGTTC	[23]
CEBPB	NM_005194	CGAAGTTGATGCAATCGGTTT	TTAAGCGATTACTCAGGGCCC	[23]
CHAC1	NM_024111	TGGTGACGCTCCTTGAAGATC	GCACTGCCTCTCGCACATT	[23]
sXBP1	NM_001079539	CTGAGTCCGCAGCAGGT	TGTCCAGAATGCCCAACAGG	
uXBP1	NM_005080	CCGCAGCACTCAGACTACG	TGTCCAGAATGCCCAACAGG	
B2M	NM_004048	ACTGAATTCACCCCCACTGA	CCTCCATGATGCTGCTTACA	[24]
GAPDH	NM_002046	CCATGTTCGTCATGGGTGTG	GGTGCTAAGCAGTTGGTGGTG	[23]
GUSB	NM_000181	GCTACTACTTGAAGATGGTGATCG	AGTTAGAGTTGCTCACAAAGGTC	
HPRT1	NM_000194	TATGCTGAGGATTTGGAAAGGG	AGAGGGCTACAATGTGATGG	[25]

DDIT3, DNA-damage-inducible transcript 3; HSPA5, heat shock 70kDa protein 5 (glucose-regulated protein, 78kDa); ATF3, activating transcription factor 3; ATF4, Activating Transcription Factor 4; MAP1LC3B, microtubule-associated protein 1 light chain 3 beta; CEBPB, CCAAT/enhancer binding protein (C/EBP), beta; CHAC1, ChaC Glutathione-Specific Gamma-Glutamylcyclotransferase 1; sXBP1, X-Box Binding Protein 1, transcript variant 2 (spliced); uXBP1, X-Box Binding Protein 1, transcript variant 1 (unspliced); B2M, Beta-2-Microglobulin; GAPDH, glyceraldehyde-3-phosphate dehydrogenase; GUSB, Glucuronidase, Beta; HPRT1, Hypoxanthine Phosphoribosyltransferase 1.

**Table 2:** Mouse qPCR primers.

Target mRNA	Accession number	Forward primer (5’-3’)	Reverse primer (5’-3’)	Ref
Ddit3	NM_007837	GAGTCCCTGCCTTTCACCTT	TTCCTCTTCGTTTCCTGGGG	
Hspa5	NM_001163434	TCCGGCGTGAGGTAGAAAAG	GGCTTCATGGTAGAGCGGAA	
Atf3	NM_007498	CTCTCACCTCCTGGGTCACT	TCTGGATGGCGAATCTCAGC	
Atf4	NM_001287180	GCAGTGTTGCTGTAACGGAC	ATCTCGGTCATGTTGTGGGG	
Cebpb	NM_009883	ACCGGGTTTCGGGACTTGA	TTGCGTCAGTCCCGTGTCCA	
Chac1	NM_026929	TATAGTGACAGCCGTGTGGG	GCTCCCCTCGAACTTGGTAT	
sXbp1	NM_001271730	CTGAGTCCGCAGCAGGT	TGTCCAGAATGCCCAAAAGG	
uXbp1	NM_013842	CCGCAGCACTCAGACTATG	TGTCCAGAATGCCCAAAAGG	
B2m	NM_009735	TGCTATCCAGAAAACCCCTCAA	GGATTTCAATGTGAGGCGGG	
Gapdh	NM_001289726	TGCCCCCATGTTTGTGATG	TGTGGTCATGAGCCCTTCC	[26]

Ddit3, Mus musculus DNA-damage inducible transcript 3; Hspa5, Mus musculus heat shock protein 5; Atf3, Mus musculus activating transcription factor 3; Atf4, Mus musculus activating transcription factor 4; Cebpb, Mus musculus CCAAT/enhancer binding protein (C/EBP), beta; Chac1, Mus musculus ChaC, cation transport regulator 1; sXbp1, Mus musculus X-Box Binding Protein 1, transcript variant 2 (spliced); uXbp1, Mus musculus X-Box Binding Protein 1, transcript variant 1 (unspliced); B2m, Mus musculus Beta-2-Microglobulin; Gapdh, Mus musculus glyceraldehyde-3-phosphate dehydrogenase.

### Detection of XBP1 mRNA splicing

Detection of *XBP1* mRNA splicing was performed by conventional PCR amplification of cDNA using primers designed upstream and downstream of the 26-nucleotides spliced sequence. Human primers were as described previously [23]. The PCR mixture contained 200 nM of each primer, 0.2 mM dNTPs, 2 mM MgCl_2_ and 1 unit of Hot Rescue DNA Polymerase (Diatheva srl), in a final volume of 50 μl. The amplification conditions were: 94°C for 7 min, 40 cycles at 94°C for 10 s, 57°C for 5 s and 72°C for 15 s. The amplification products of the unspliced and spliced forms were 137 bp and 111 bp, respectively. Murine *Xbp1* was amplified with primers 5’-ACACGCTTGGGAATGGACAC-3’ (forward) and 5’-CCATGGGAAGATGTTCTGGG-3’ (reverse) with the following conditions: 94°C for 7 min, 40 cycles at 94°C for 10 s, 58°C for 10 s and 72°C for 20 s. The amplification products of the murine unspliced and spliced forms were 171 bp and 145 bp, respectively. The PCR products were separated on 3.5% agarose gel and visualized with GelRed^™^ (Biotium, Hayward, CA). GeneRuler 100-bp Plus DNA Ladder (Thermo Scientific, Waltman, MA, USA) was included on the gels as a size standard.

The relative amount of *uXBP1* and *sXBP1* were also monitored by qPCR using primer pairs specific for the spliced and unspliced form (Tables 1 and 2), with the following amplification conditions: 95°C for 10 min, 40 cycles at 95°C for 15 s and 60°C for 50 s.

### Western immunoblot analysis

U937 and THP-1 derived macrophage-like cells were processed for Western blot analysis as previously reported [27]. Briefly, cells were directly harvested in 20 mM Hepes pH 7.9, 25% glycerol, 0.42 M NaCl, 1.5 mM MgCl_2_, 0.2 mM EDTA, 1 mM DTT, 1 mM Naf, 1 mM Na_3_VO_4_, and 1X complete protease inhibitor cocktail (Roche Diagnostics Ltd.,Mannheim, Germany). After an incubation of 20 min on ice, the samples were frozen and thawed twice and whole cell extract was collected after centrifugation at 10,000 x g for 10 min. Total cell lysates were fractionated by SDS-PAGE, and gels were electroblotted onto a nitrocellulose membrane (0.2 μm pore size) (Bio-Rad Laboratories, Inc., Hercules, CA, USA). The resulting blots were probed with the following primary antibodies: anti-phospho-Akt (9271), anti-Akt (9272), anti-Cleaved Caspase-3 (9661) and anti-CHOP (2895), purchased from Cell Signalling Technology (Beverly, MA, USA); anti-XBP1 (M-186:sc-7160), anti-GRP78/HSPA5 (sc-166490) and anti-phospho-eIF2α (sc-101670) purchased from Santa Cruz Biotechnology Inc. (Santa Cruz, CA, USA); anti-actin (A2066) purchased from Sigma-Aldrich. Signals were detected using horseradish peroxidase-conjugated secondary antibodies (Bio-Rad Laboratories Inc.). Blots were treated with chemiluminescence reagents (ECL Kit, Amersham Bioscience, Arlington Heights, IL, USA), and the immunoreactive bands were detected with light-sensitive film (Amersham Hyperfilm ECL, GE Healthcare Life Sciences) and quantified by Chemi-Doc System (Bio-Rad Laboratories, Inc.) equipped with Quantity One software.

### Statistical analysis

Data were analyzed by non-parametric test (Wilcoxon signed-rank test or Mann-Whitney test) or by two-way ANOVA followed by Bonferroni post hoc test respectively for gene expression and western blotting, using Prism software (GraphPad, San Diego, CA, USA). All data are presented as the mean ± standard error of the mean (SEM).

### Ethics statement

Housing and treatment of mice were in compliance with the recommendations in the Guide for the Care and Use of Laboratory Animals by the Health Ministry, law 116, 1992. Experiments were approved by the Committee on the Ethics of Animal Experiments of the University of Urbino “Carlo Bo”. The animals were euthanized by carbon dioxide.

## Results

### *L*. *infantum* infection induces Akt phosphorylation and inhibits the tunicamycin-induced caspase-3 activation in U937-derived macrophages

Previously, the Akt phosphorylation at Ser473 and the inhibition of caspase-3 activation after treatment with camptothecin or actinomycin D have been reported in RAW 264.7 macrophages infected with promastigotes of *L*. *major*, *L*. *amazonensis* and *L*. *pifanoi* [6]. Moreover, it was also shown that *L*. *infantum* affected the survival of U937 cells via a mechanism involving inhibition of caspase-3 activation after treatment with actinomycin D [5].

To further investigate the mechanisms underlying the apoptosis resistance in parasitized cells, we first determined whether *L*. *infantum* infection was able to activate the Akt signaling pathway and inhibit caspase-3 cleavage in our infection model, using tunicamycin (an inhibitor of N-glycosylation) as ER stressor and inducer of apoptosis.

**Fig 1:**
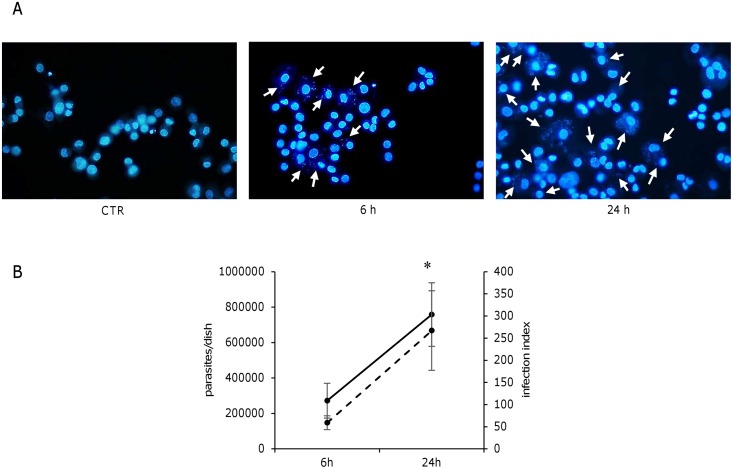
*L*. *infantum* actively infects U937-derived macrophages. A) U937-derived macrophages were infected with *L*. *infantum* promastigotes; after 6 h or 24 h cells were washed, stained with Hoechst dye and observed with a fluorescence microscope to monitor internalized parasites. Arrows indicate parasitized cells. B) Active infection was monitored by calculating infection index, considering at least 300 total macrophages per infection time (dotted line), and by a qPCR assay targeting kDNA of *Leishmania* (solid line). Both methods allowed to detect a significant increase in infection from 6 h to 24 h. The data are representative of three independent experiments. CTR, uninfected control. * p < 0.05.

**Fig 2:**
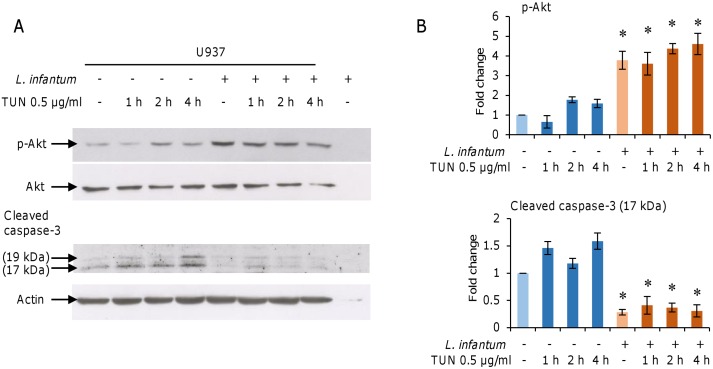
*L*. *infantum* infection induces cell survival and apoptosis inhibition. U937-derived macrophages were infected with *L*. *infantum* promastigotes for 18 h. Infected and non-infected cells were treated with 0.5 μg/ml tunicamycin for 1 h, 2 h and 4 h. A) The Akt phosphorylation and cleavage of caspase-3 were analyzed in total cell lysates by western blotting. B) Band density quantification was performed using a Chemi-Doc System. Results are representative of three experiments. * p < 0.05, two-way ANOVA followed by Bonferroni post hoc test.

The U937-derived macrophages were infected with promastigotes as indicated in methods. The infection index was 59 ± 15.7 and 266.8 ± 89.8 after 6 h and 24 h of infection, respectively (average ± SD values from 3 independent experiments). Active infection was confirmed also by quantification of *Leishmania* parasites into cells using a previously developed qPCR assay [21] (Fig 1). After 18 h infection, Akt phosphorylation and cleavage of caspase-3 were assessed by western blotting. A significant increase in Akt phosphorylation was observed in infected cells compared to uninfected controls. Tunicamycin treatment did not significantly affect Akt phosphorylation, neither in infected nor in uninfected cells (Fig 2). Moreover, the cleavage of caspase-3, which was evident in cells treated with tunicamycin, was completely prevented in infected cells (Fig 2). Taken together, these data confirmed the establishment of a productive infection and were in agreement with previous observations obtained with the apoptosis inducers camptothecin and actinomycin D.

### *L*. *infantum* infection induces mild ER stress response

**Fig 3:**
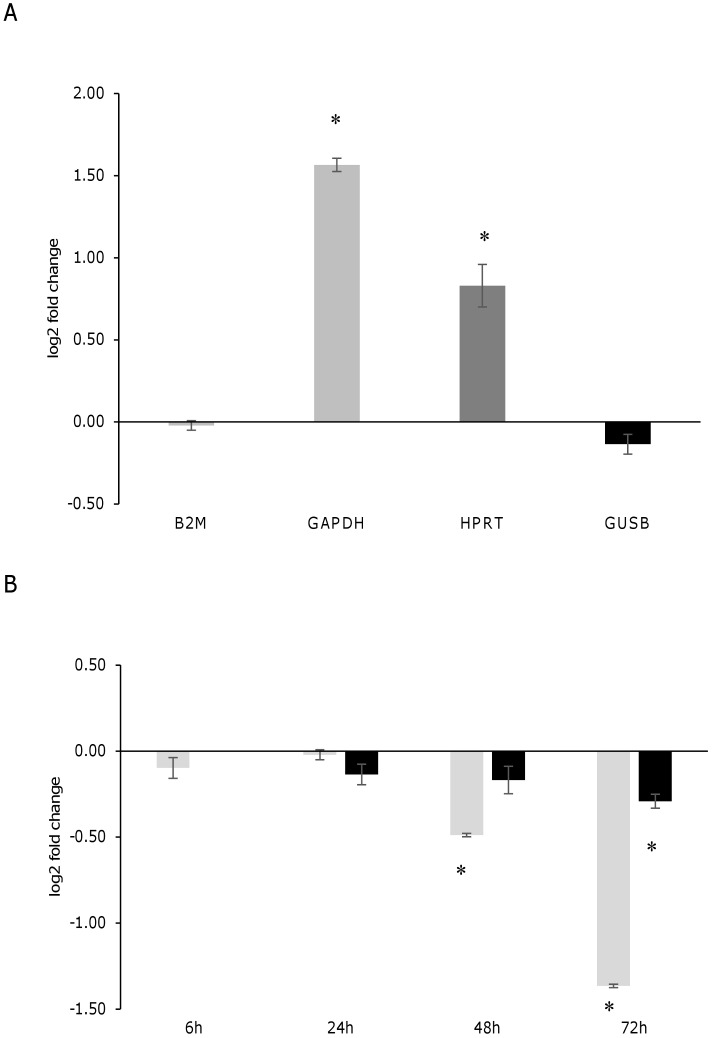
Evaluation of the reference genes. A) Log2 fold change of the four candidate reference genes (*B2M*, *GAPDH*, *HPRT*, *GUSB*) in U937-derived macrophages after 24 h infection in comparison to the non-infected controls. B) Evaluation of *B2M* (grey bars) and *GUSB* (black bars) expression in U937-derived macrophages infected for 6 h, 24 h, 48 h, 72 h, in comparison to the non-infected controls. The amount corresponding to 50 ng of total RNA used for cDNA synthesis was loaded per each PCR tube. * p < 0.05.

**Fig 4:**
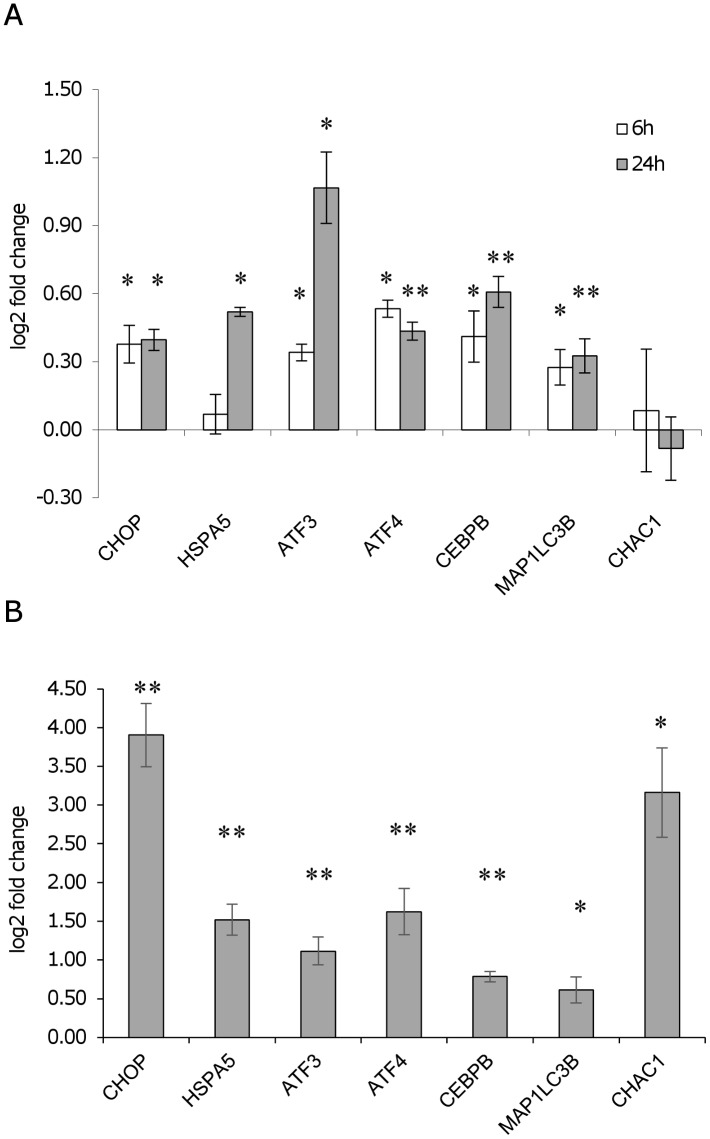
ER stress expression markers in infected U937-derived macrophages. A) Gene expression profiling in U937-derived macrophages 6 h and 24 h after infection with *L*. *infantum*. The graph shows the log2 fold changes in comparison to the control (non-infected). B) Gene expression in U937-derived macrophages following tunicamycin treatment (0.5 μg/ml, 6 h). The graph shows the log2 fold changes in comparison to the control (DMSO) values. Data are represented as the mean ± SEM of at least three experiments. * p < 0.05, ** p < 0.01.

**Fig 5:**
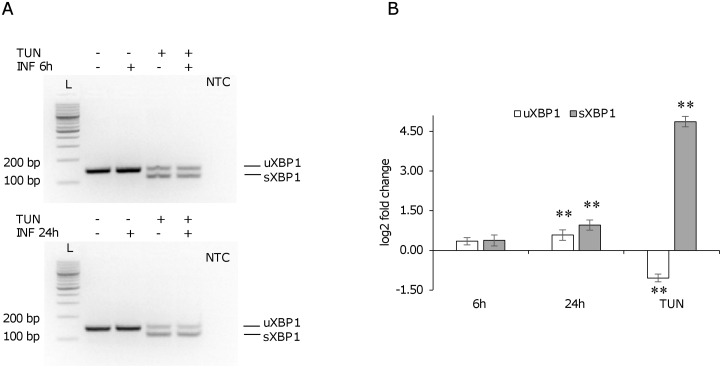
*XBP1* induction following *L*. *infantum* infection in U937-derived macrophages. U937-derived macrophages were infected with *L*. *infantum* promastigotes for 6 h or 24 h. As positive control for *XBP1* splicing/induction, infected and non-infected cells were treated with 0.5 μg/ml tunicamycin for 6 h. A) *XBP1* splicing was not detected using conventional PCR in infected cells. B) A significant induction of both uXBP1 and sXBP1 was shown after 24 h of infection by qPCR, while tunicamycin treatment induced an increase in sXBP1 and a decrease in the uXBP1. ** p < 0.01. TUN, tunicamycin; L, 100 bp DNA ladder; NTC, no template control.

To investigate the ER stress response in U937-derived macrophages infected with *L*. *infantum*, the expression of ER stress marker genes indicated in Table 1 was monitored by qPCR at 6 h and 24 h post-infection. The reference genes *B2M* and/or *GUSB* were selected since their expression did not change significantly following infection (Fig 3A). Later time points were not taken into consideration since the expression of the reference genes diminished significantly starting from 48 h post-infection (Fig 3B). Moreover, a general downregulation of gene expression after 72 h infection has been reported in literature [28,29]. The gene expression analysis showed a significant induction of the ER stress markers *DDIT3/CHOP*, *ATF3*, *ATF4*, *CEBPB* and the autophagy marker *MAP1LC3B*, both at 6 h and 24 h post-infection; *HSPA5* was significantly induced only after 24 h, while *CHAC1* did not show any significant variation (Fig 4A). A similar trend of gene expression was detected after infection with two *L*. *infantum* isolates (S1 Fig). As positive control of UPR, cells were treated with the ER stress inducer tunicamycin (0.5 μg/ml). All genes tested resulted significantly upregulated after tunicamycin treatment (Fig 4B). In this case, the magnitude of induction was significantly higher as compared with infected cells (p < 0.05, Wilcoxon matched-pairs signed-rank test). Also treatment with DTT induced a significant upregulation of ER stress markers, although to a lesser extent (S2 Fig). The splicing of *XBP1* mRNA was not detected in infected cells using conventional PCR (Fig 5A). However, with a qPCR assay designed to amplify specifically the spliced and the unspliced sequence, a significant increase in the spliced form was revealed after 24 h of infection (Fig 5B). Interestingly, in contrast to the canonical UPR induced by tunicamycin, also uXBP1 expression increased significantly, although to a less extent compared to sXBP1.

**Fig 6:**
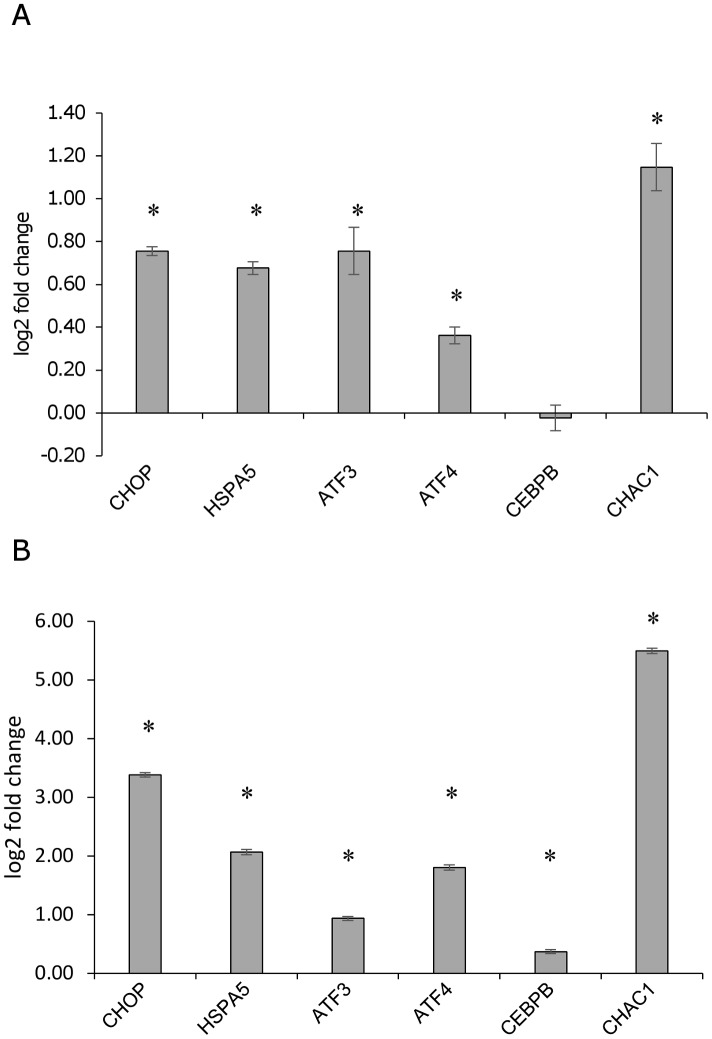
ER stress expression markers in infected murine macrophages. A) Gene expression profiling in murine macrophages 24 h after infection with *L*. *infantum*. The graph shows the log2 fold changes in comparison to the control (non-infected). B) Gene expression in murine macrophages following tunicamycin treatment (2 μg/ml, 4 h). The graph shows the log2 fold changes in comparison to the control (DMSO) values. Data are represented as the mean ± SEM of two experiments, * p < 0.05.

**Fig 7:**
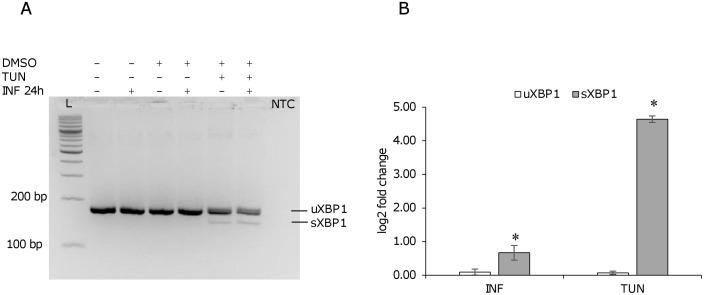
*Xbp1* induction following *L*. *infantum* infection in murine macrophages. Murine macrophages were infected with *L*. *infantum* promastigotes for 24 h. As positive control for *Xbp1* splicing/induction, infected and non-infected cells were treated with 2 μg/ml tunicamycin for 4 h. A) *Xbp1* splicing was not detected using conventional PCR in infected cells. B) A significant induction of sXbp1 was shown after 24 h infection or tunicamycin treatment by qPCR. ** p < 0.01. TUN, tunicamycin; L, 100 bp DNA ladder; NTC, no template control.

A similar trend of gene expression was observed in murine macrophages at 24 h post-infection (Figs 6 and 7) (infection index 83 ± 33) with few differences regarding the expression of *Chac1* and *Cebpb* (Fig 6A). Moreover, the unspliced form of *Xbp1* did not appear significantly affected neither by infection nor by tunicamycin treatment (Fig 7B).

**Fig 8:**
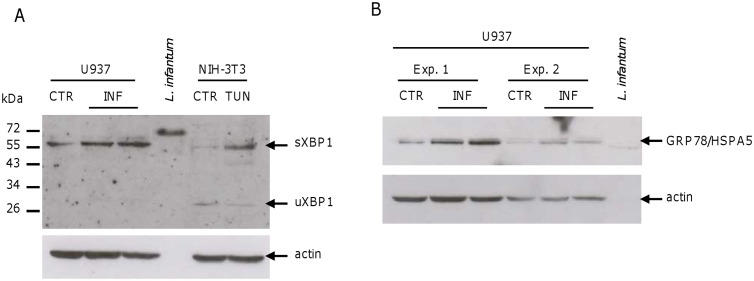
*L*. *infantum* infection induces sXBP1 and GRP78/HSPA5 proteins. U937-derived macrophages were infected with *L*. *infantum* promastigotes for 18 h. A) The sXBP1 protein levels were analyzed in total cell lysates by western blotting. Infected cells are shown in duplicate. NIH-3T3 cells treated with tunicamycin were used as positive control for antibody signal. B) The HSPA5/GRP78 protein levels were analyzed in total cell lysates by western blotting. Two experiments are shown. Infected cells are shown in duplicate.

**Fig 9:**
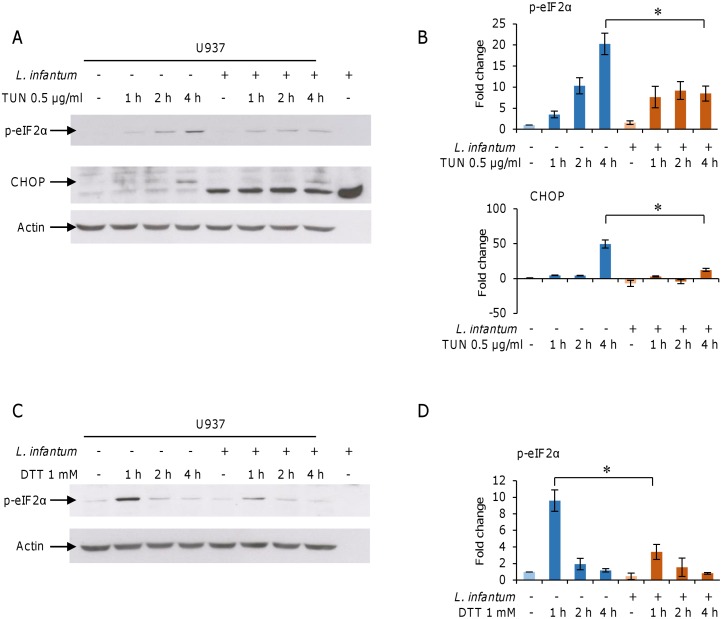
*L*. *infantum* inhibits tunicamycin- and DTT-induced ER stress. U937-derived macrophages were infected with *L*. *infantum* promastigotes for 18 h. Infected and non-infected cells were treated with 0.5 μg/ml tunicamycin (A, B) or 1 mM DTT (C, D) for 1 h, 2 h and 4 h. A, C) The phosphorylation of eIF2α and DDIT3/CHOP protein levels were analyzed in total cell lysates by western blotting. B, D) Band density quantification was performed using a Chemi-Doc System. Results are representative of three experiments. * p < 0.05, two-way ANOVA followed by Bonferroni post hoc test. TUN, tunicamycin.

The western blot analyses of U937-derived macrophages after *L*. *infantum* infection showed the induction of sXBP1 and GRP78/HSPA5 proteins (Fig 8). However, *L*. *infantum* infection did not appear to induce the ER stress markers phospho-eIF2α and DDIT3/CHOP (Fig 9). Taken together, these results account for a mild induction of ER stress and UPR following *L*. *infantum* infection.

### *L*. *infantum* infection delays/attenuates the effects of the ER stressors tunycamycin and DTT

Since mild ER stress has been shown to have a protective role in cell lines and disease models [30–32], we investigated the ability of *L*. *infantum* to modulate the UPR in infected cells treated with ER stress inducers. To this end, U937 and THP1-derived macrophages were infected with promastigotes for 14 h, treated with 0.5 μg/ml tunicamycin or 1 mM DTT and harvested after 1 h, 2 h and 4 h. The phosphorylation of eIF2α and DDIT3/CHOP protein levels were assessed by western blotting.

In U937-derived macrophages, tunicamycin induced a time-dependent eIF2α phosphorylation and DDIT3/CHOP expression. Interestingly, after 4 h treatment with tunicamycin, a significant reduction in both phospho-eIF2α and DDIT3/CHOP protein levels was detected in infected cells compared to non-infected cells (Fig 9A and 9B), suggesting an attenuation of host UPR by *L*. *infantum* infection. The treatment with DTT induced eIF2α phosphorylation as tunicamycin, although at earlier time. Also in this case, the eIF2α phosphorylation was significantly reduced by *L*. *infantum* infection (Fig 9C and 9D). These results were confirmed in THP1-derived macrophages: in fact, also in these cells *L*. *infantum* infection reduced significantly the tunicamycin-induced Eif2α phosphorylation and DDIT3/CHOP expression (S3 Fig).

**Fig 10:**
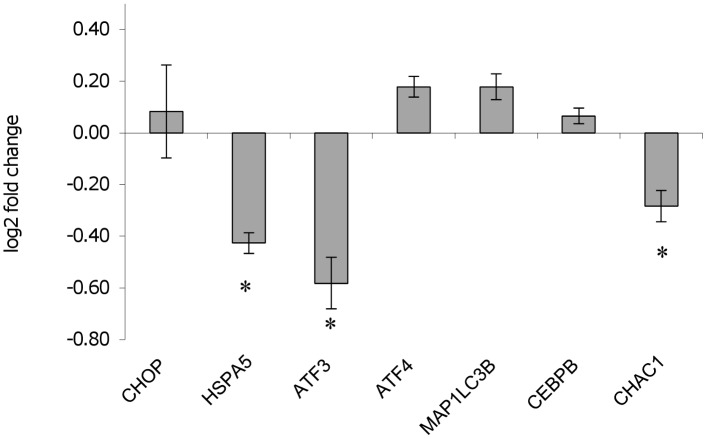
*L*. *infantum* inhibits the expression of tunicamycin-induced ER stress marker genes. U937-derived macrophages were infected with *L*. *infantum* promastigotes for 18 h. Infected and non-infected U937-derived macrophages were treated with 0.5 μg/ml tunicamycin for 2 h. The graph shows the log2 fold changes of ER stress marker genes induced by tunicamycin in infected cells compared to the non-infected cells. * p < 0.05.

The expression of ER stress marker genes was also evaluated by qPCR in non-infected and infected U937-derived macrophages treated for two hours with tunicamycin or DTT. Following tunicamycin treatment, the induction of the ER stress marker genes *HSPA5*, *ATF3* and *CHAC1* was significantly lower in infected cells, compared to non-infected cells (Fig 10). On the other hand, the induction of ER stress markers following DTT treatment did not change significantly between infected and uninfected cells (not shown).

## Discussion

*Leishmania* is a pathogenic protozoan that infects about one million people per year worldwide. This parasite replicates inside macrophages and has evolved strategies to subvert host defense mechanisms such as ROS and NO production, antigen presentation, immune activation and apoptosis [1]. Some of these strategies have been investigated using different *Leishmania* species and infection models. For example, *L*. *amazonensis* (belonging to *L*. *mexicana* complex which causes cutaneous leishmaniasis) suppresses NO production by repressing the iNOS expression via the NF-kB transcription factor [33] and reduces ROS accumulation by inducing SOD-1 [34] in human and mouse cells. Moreover, it has been shown that *L*. *major* downregulates protein synthesis in murine macrophages by GP63-mediated cleavage of the mammalian target of rapamycin (mTOR), a serine/threonine kinase that regulates the translational repressor 4E-BP1 [35]. ER stress also induces a reduction in mTOR activity, which in turn leads to hypophosphorylation of 4E-BP1 and translation repression [36]. Moreover, 4E-BP1 is also a target of the UPR-induced transcription factor ATF4 [37]. Several intracellular pathogens are known to induce ER stress and activate the UPR in the host cell. However, little is known about ER stress and UPR in macrophages infected by *Leishmania* and its role in the pathogenesis.

In this study, we firstly confirmed previous findings (*i*.*e*. induction of Akt phosphorylation and inhibition of caspase-3 activation) in our infection model, using tunicamycin as apoptosis inducer. Tunicamycin triggers apoptosis via induction of ER stress, while camptothecin and actinomycin D (used in previous studies) are inhibitors of DNA topoisomerase I and DNA replication/transcription, respectively. Then, we showed that *L*. *infantum* infection was associated with a mild UPR response in human and murine macrophages. This response included the upregulation of several ER stress marker genes, which was much lower respect to the one observed after treatment with the ER stressor tunicamycin, accounting for an averagely moderate ER stress response in the cell population. The fact that not all cells were simultaneously infected and/or the different kinetic/activation of the three UPR branches could contribute to explain the induction of some ER stress marker proteins (i.e. GRP78/HSPA5 and sXBP1) and the lack of induction of others (i.e. phospho-eIF2α and DDIT3/CHOP). In fact, it is possible that *L*. *infantum* infection perturbs ER homeostasis in a moderate manner that it cannot be detected by analysis of selected marker proteins in a heterogeneous pool of cells. Alternatively, specific branches of UPR could be preferentially activated at the infection time analyzed.

Few differences were evidenced between infected cells and cells treated with tunicamycin: *CHAC1* expression did not change significantly in U937-derived macrophages infected with *L*. *infantum* reference strain or isolates, while it was strongly upregulated after tunicamycin treatment. On the other hand, in infected murine macrophages *Chac1* was significantly upregulated while *Cebpb* expression did not change. These differences could be explained by some heterogeneity of response rate in different host cells and/or by different infection rates.

Concerning the monitoring of *XBP1* splicing in infected cells, the *sXBP1* form was not detected with conventional PCR neither in human nor in mouse macrophages. However, a qPCR assay allowed to detect a significant induction of the *sXBP1* form in both human and mouse cells after 24 h infection. Interestingly, in human cells also the *uXBP1* form was significantly upregulated, unlike in the canonical ER stress induced by tunicamycin (Fig 5). It is known that the expression of HO-1 can be mediated by PERK [38] and uXBP1 [11] via NRF2 pathway; notably, the induction of HO-1 has been demonstrated in *L*. *chagasi* infection and it has been associated with diminished production of TNF-α and ROS and enhanced parasite survival [39]. Our findings may suggest the induction/modulation of ER stress as a mechanism by which *Leishmania* triggers host antioxidant enzymes acting as scavengers of superoxide anions generated during infection. Recently, the activation of XBP1 splicing arm of the ER stress response has been reported in a murine macrophage cell line infected with *L*. *amazonensis* [40]. The confirmation of this finding, although with some differences, in our infection models suggests some common pathogenic mechanism between cutaneous and visceral species.

Although it is clear that severe or prolonged ER stress can lead to cell death, low levels of ER stress may be beneficial to cells by eliciting a mild (adaptive) UPR that “prepares” the cell, increasing the cellular resistance to subsequent ER stress; this process, called ER hormesis, has been extensively reviewed by Mollereau et al [41]. In particular, both the IRE1-XBP1 and PERK-ATF4 arms of the UPR contribute to the ER hormesis. In this view, the mild UPR triggered by *L*. *infantum* infection can be seen as a preconditioning or adaptive stress response (*i*.*e*. hormesis). In fact, the effects of ER stress inducers were attenuated in both U937 and THP1-derived macrophages infected by *L*. *infantum*. The differences in gene expression observed after treatment of infected cells with the ER stress inducer tunicamycin (a glycosilation inhibitor) and DTT (a disulfide bond-reducing agent) may be due to differential activation kinetics of ER stress signaling [42]. In fact, gene expression was monitored after two hours treatment, but DTT acts earlier than tunicamycin (phospho-eIF2α was evident after 1 h).

The cellular signaling pathways and molecular mechanisms that mediate hormetic responses typically involve transcription factors such as NRF2 and NF-κB. As a result, cells increase their production of cytoprotective proteins including phase 2 and antioxidant enzymes, and protein chaperones [43]. Therefore, the resistance to apoptosis of infected cells reported in literature could be due to ER hormesis induced by *Leishmania* in the early phases (first 24 h) of infection. The increased resistance delays cell death, allowing parasite transformation into amastigotes and replication. Lastly, *Leishmania* parasites exploits the apoptotic host cell to spread to neighboring macrophages, reducing the exposure to extracellular immune recognition system [44]. The mild UPR in infected cells could also contribute to explain many previous findings concerning manipulation of host cellular pathways, such as induction of antioxidant enzymes, translation repression, induction of autophagy, and also Akt phosphorylation, which is induced by ER stress in a PERK-dependent manner [45].

*Leishmania* could induce a mild UPR in infected host cells due to moderate “physiological” perturbation of ER homeostasis, therefore leading to hormesis. Alternatively, the parasite could actively modulate (at the transcriptional or post-transcriptional level) the host UPR, curbing it to a mild response and consequently inducing a protective response to prolong host cell survival. This aspect will need further investigations.

In conclusion, the mild ER stress response elicited by *Leishmania* infection could represent an upstream pathogenic mechanism common among different *Leishmania* (*Leishmania*) species, and could be part of the strategies, together with known virulence factors (e.g. GP63), that *Leishmania* has evolved to survive/replicate into host cell. In this view, the UPR may also represent a target for new therapeutic approaches since current drug treatments have toxic side effects and lead to drug resistance.

## Supporting Information

S1 Fig ER stress expression markers in U937-derived macrophages infected with two *L*. *infantum* isolates. Gene expression profiling in U937-derived macrophages 24 h after infection with *L*. *infantum* isolate 1 (blue bars) and isolate 2 (orange bars). The graph shows the log2 fold changes in comparison to the control (non-infected). Data are represented as the mean ± SEM of two experiments. * p < 0.05.
(PPTX)

S2 Fig ER stress expression markers in U937-derived macrophages treated with DTT. Gene expression in U937-derived macrophages following 2 h treatment with 1 mM DTT. The graph shows the log2 fold changes in comparison to the control (mean ± SEM).
(PPTX)

S3 Fig *L*. *infantum* inhibits tunicamycin-induced ER stress in THP1-derived macrophages. THP1-derived macrophages were infected with *L*. *infantum* promastigotes for 18 h. Infected and non-infected cells were treated with 0.5 μg/ml tunicamycin for 1 h, 2 h and 4 h. The phosphorylation of eIF2α and DDIT3/CHOP protein levels were analyzed in total cell lysates by western blotting. TUN, tunicamycin.
(PPTX)
