# Supplementary figures and images for: Leishmania infantum Induces Mild Unfolded Protein Response in Infected Macrophages
Source: PLoS One. 2016 Dec 15;11(12):e0168339. doi: 10.1371/journal.pone.0168339 (PMC5158320; doi:10.1371/journal.pone.0168339)

## Slide 1
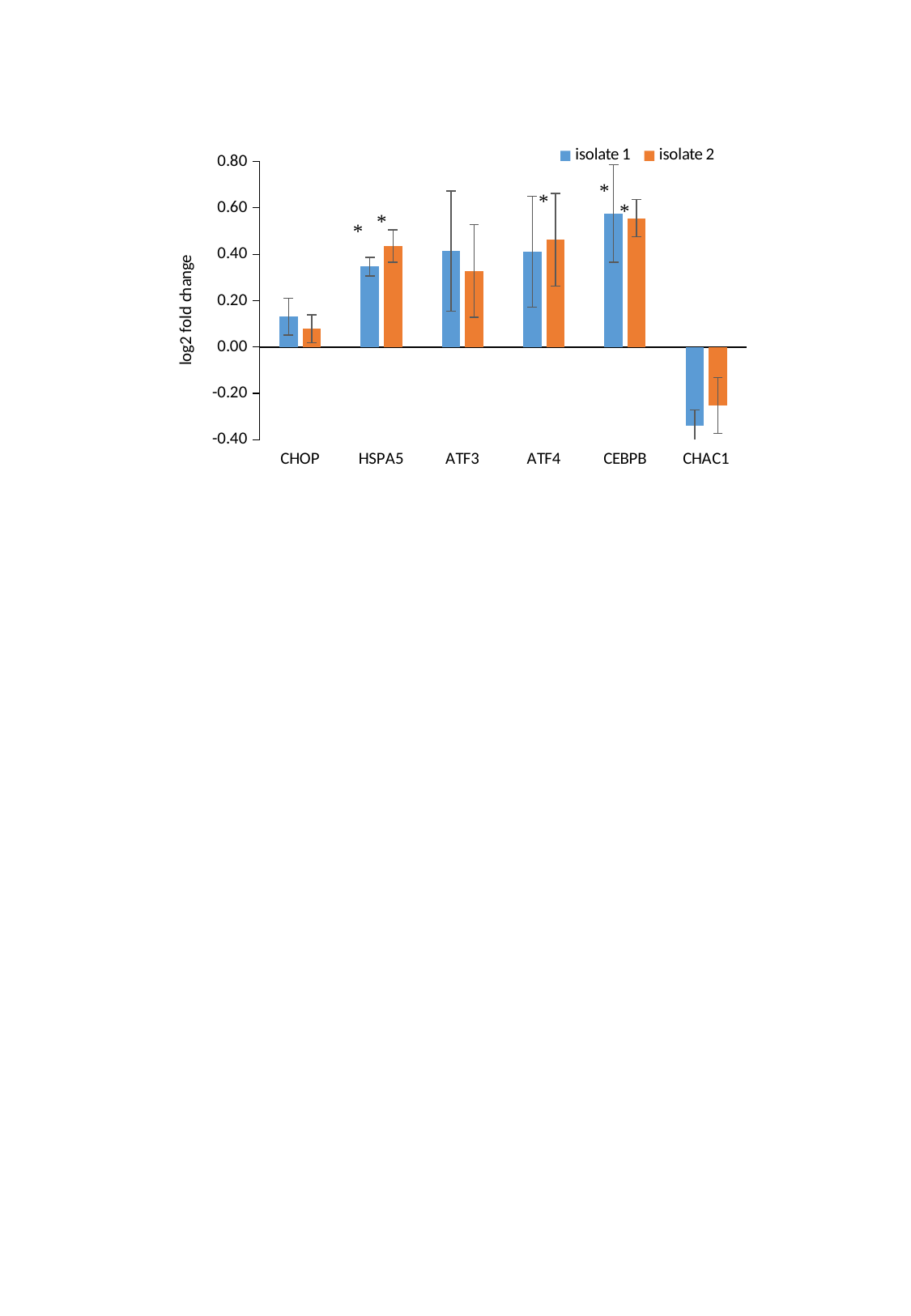

### Chart
| Category | isolate 1 | isolate 2 |
|---|---|---|
| CHOP | 0.1311597848506158 | 0.07794773650957132 |
| HSPA5 | 0.3467495846017695 | 0.43541493817989596 |
| ATF3 | 0.4139323103649882 | 0.32856844777953725 |
| ATF4 | 0.4110877201591663 | 0.46331462627671044 |
| CEBPB | 0.5765534838565252 | 0.5557408208249434 |
| CHAC1 | -0.34082941210168005 | -0.2523337234067754 |*
*
*
*
*

Supplement: S1 Fig — Gene expression profiling in U937-derived macrophages 24 h after infection with L. infantum isolate 1 (blue bars) and isolate 2 (orange bars). The graph shows the log2 fold changes in comparison to the control (non-infected). Data are represented as the mean ± SEM of two experiments. * p < 0.05. (PPTX) [file pone.0168339.s001.pptx]

## Slide 1
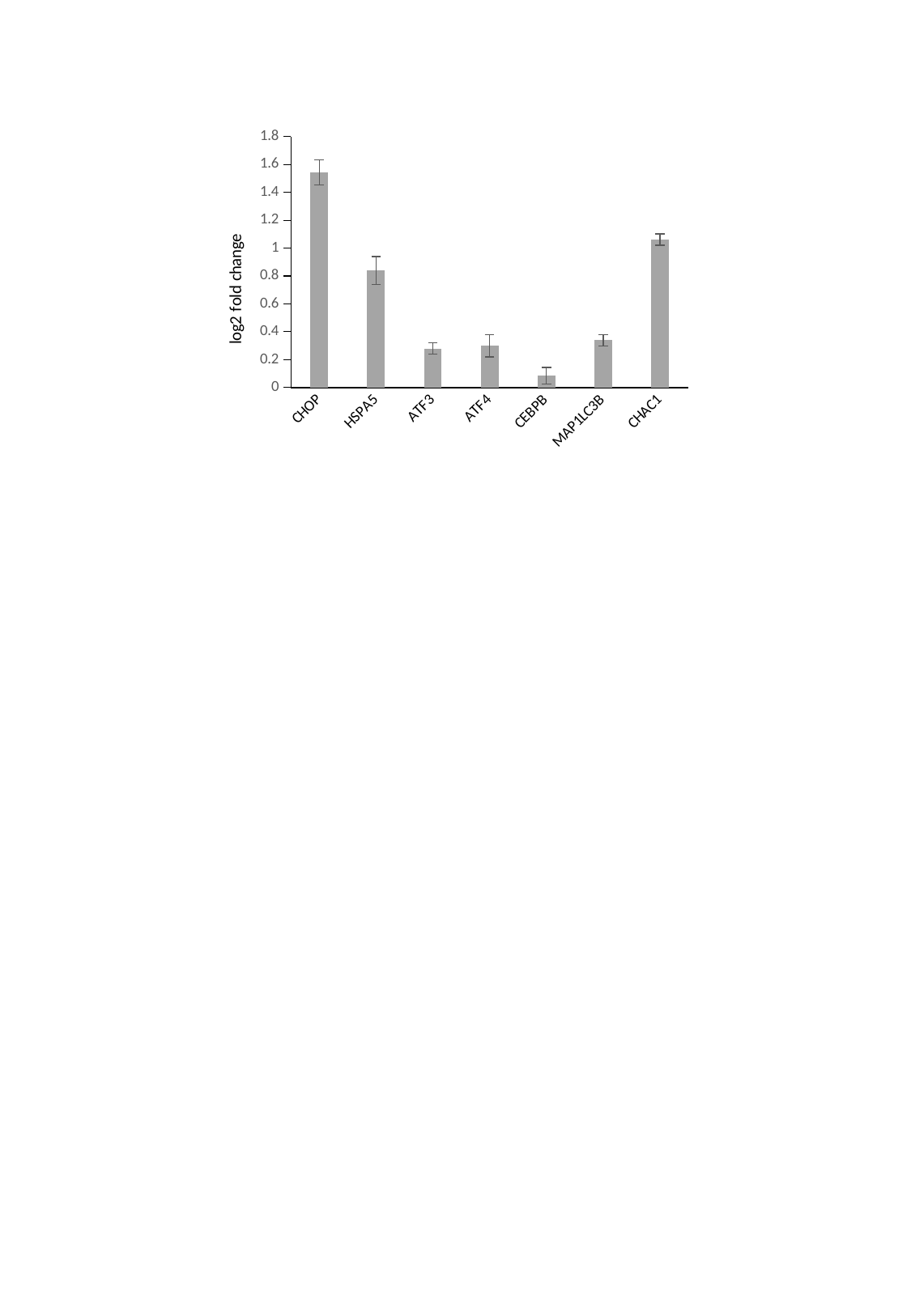

### Chart
| Category | |
|---|---|
| CHOP | 1.5418807845971771 |
| HSPA5 | 0.8396273687038399 |
| ATF3 | 0.28014234231867513 |
| ATF4 | 0.29865831556451516 |
| CEBPB | 0.08406426478847455 |
| MAP1LC3B | 0.3385849694091225 |
| CHAC1 | 1.0614306944416474 |

Supplement: S2 Fig — Gene expression in U937-derived macrophages following 2 h treatment with 1 mM DTT. The graph shows the log2 fold changes in comparison to the control (mean ± SEM). (PPTX) [file pone.0168339.s002.pptx]

## Slide 1
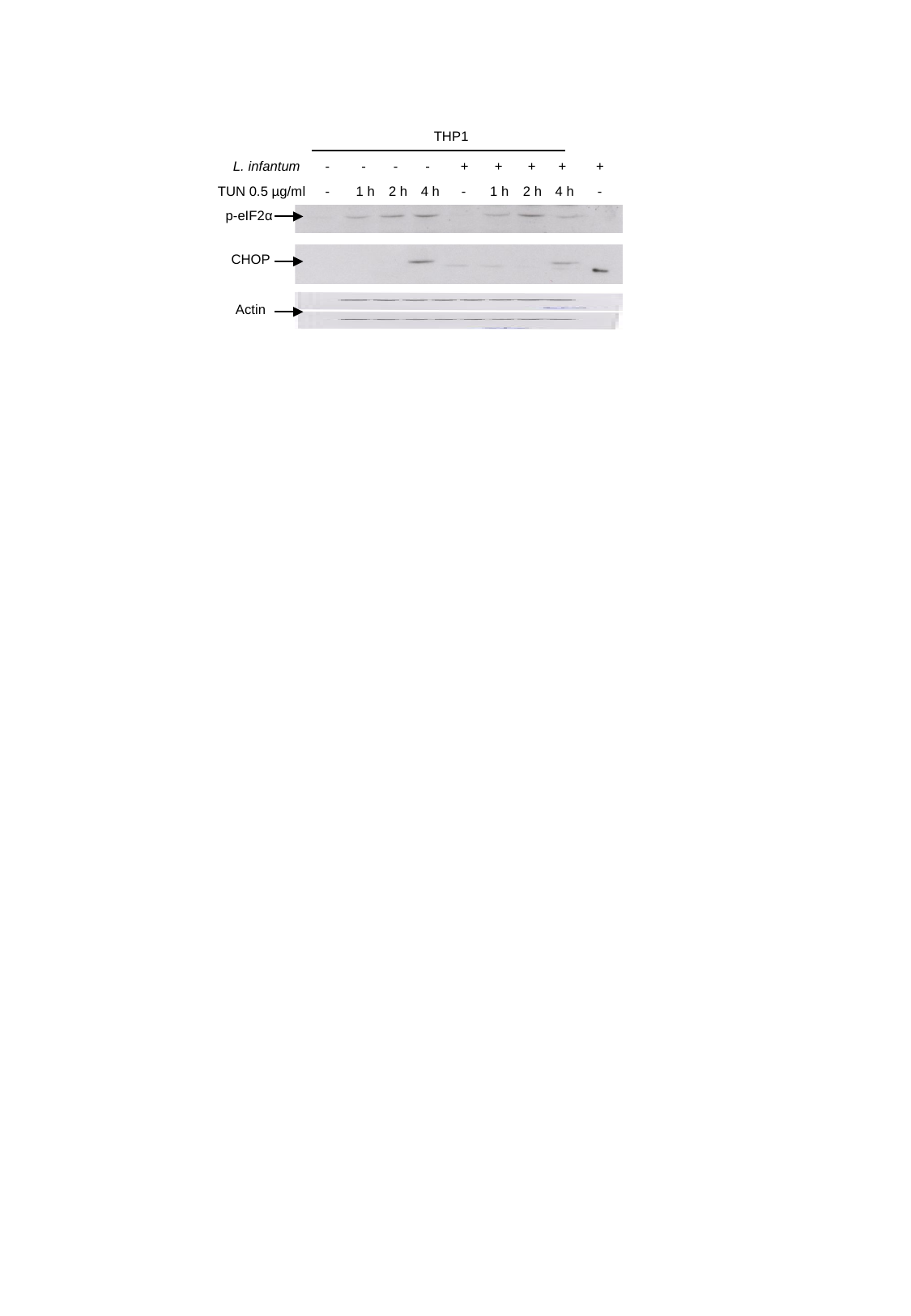

THP1
-
-
-
-
+
+
+
+
+
L. infantum
TUN 0.5 µg/ml
-
1 h
2 h
4 h
-
1 h
2 h
4 h
-
p-eIF2α
CHOP
Actin

Supplement: S3 Fig — THP1-derived macrophages were infected with L. infantum promastigotes for 18 h. Infected and non-infected cells were treated with 0.5 μg/ml tunicamycin for 1 h, 2 h and 4 h. The phosphorylation of eIF2α and DDIT3/CHOP protein levels were analyzed in total cell lysates by western blotting. TUN, tunicamycin. (PPTX) [file pone.0168339.s003.pptx]
